# Patterns of adenoid and tonsil growth in Japanese children and adolescents: A longitudinal study

**DOI:** 10.1038/s41598-018-35272-z

**Published:** 2018-11-20

**Authors:** Takayoshi Ishida, Asuka Manabe, Shin-Sheng Yang, Hyung Sik Yoon, Eiichiro Kanda, Takashi Ono

**Affiliations:** 1 Department of Orthodontic Sciences, Tokyo Medical Dental University Graduate School, Tokyo, Japan; 2 All Barun Dental Clinic, Suwon, Korea; 3 Medical Science, Kawasaki Medical School, Kawasaki Medical School, Okayama, Japan

## Abstract

Lymphoid tissues, such as adenoids (Ad) and tonsils (Tn), are suggested to undergo hypertrophy during childhood and involution in adulthood. Enlargement of Ad and Tn can cause transient obstruction of the respiratory airways, thus inducing obstructive sleep apnoea. To date, the standard Ad and Tn sizes have not been reported, and there are no explicit objective criteria for evaluating their sizes or deducing whether they have enlarged, reduced, or remained constant over time. Our previous cross-sectional study revealed the age-dependent airway occupation ratio of Ad and Tn in Japanese individuals. We conducted a longitudinal observational study of the Ad and Tn sizes in Japanese individuals aged 6–20 years. Ninety individuals were retrospectively enrolled. The average and standard deviation of the sizes was calculated in 5 age-based groups.

## Introduction

Approximately 80 years ago, Scammon described 4 types of growth curves (lymphoid, neural, general, and genital) that represent human growth; this model is still used in various fields. Among these types, the lymphoid type has a unique growth pattern. Organs belonging to the lymphoid type, such as the adenoids (Ad) and tonsils (Tn), attain approximately 200% growth by late childhood and then involute during adulthood^1^.

However, in clinical orthodontics, many adults who have enlarged Ad and Tn, which have not undergone involution, are encountered. In patients with respiratory disorders, such as obstructive sleep apnoea (OSA), enlarged Ad and Tn have been reported to confer a risk of various diseases, such as endocrine abnormalities, cerebral vascular disturbances, digestive disorders, diabetes mellitus, abnormal lipid metabolism, a funnel chest, an adenoid face, short height, and low weight^2–8^.

Tonsillectomy and adenoidectomy are common and effective treatments for improving respiratory impairment caused by enlarged Ad and Tn. Indications for these surgical procedures vary across countries, but upper airway obstruction due to hypertrophy of the lymphatic tissue in the Waldeyer’s ring, which includes Ad and Tn, is one indication^9–14^.

To date, no longitudinal observational study has assessed the sizes of the Ad and Tn. Therefore, it remains unclear whether hypertrophy of the Ad and Tn occurs, whether the hypertrophy is followed by involution, and whether surgery is required in such cases. Orthodontists are often confronted with patients in whom hypertrophy of the Ad and Tn is suspected, however, many of them do not use endoscope in daily clinical practice, thus cannot diagnose the enlarged Ad and Tn. Endoscopic examination is appropriate for diagnosing hypertrophy of the Ad and Tn, which is one of the risk factors for OSA, while polysomnography (PSG) is the gold standard for diagnosing OSA, but still expensive and time-consuming. Therefore, a credible and simple method for assessing the size of the Ad and Tn may be required.

Therefore, a credible and simple method for assessing the sizes of the Ad and Tn is required.

In our previous cross-sectional study, we determined the average Ad/nasopharynx (Np) and Tn/oropharynx (Op) ratios in Japanese children and teenagers. We performed cephalometric analysis, a highly reproducible examination that is routinely performed in orthodontic assessments^15–17^. The present study aimed to construct growth curves for the Ad and Tn, compare these growth curves, and provide a method to assist physicians in deciding whether to perform adenotonsillectomy.

## Results

**Figure 1:**
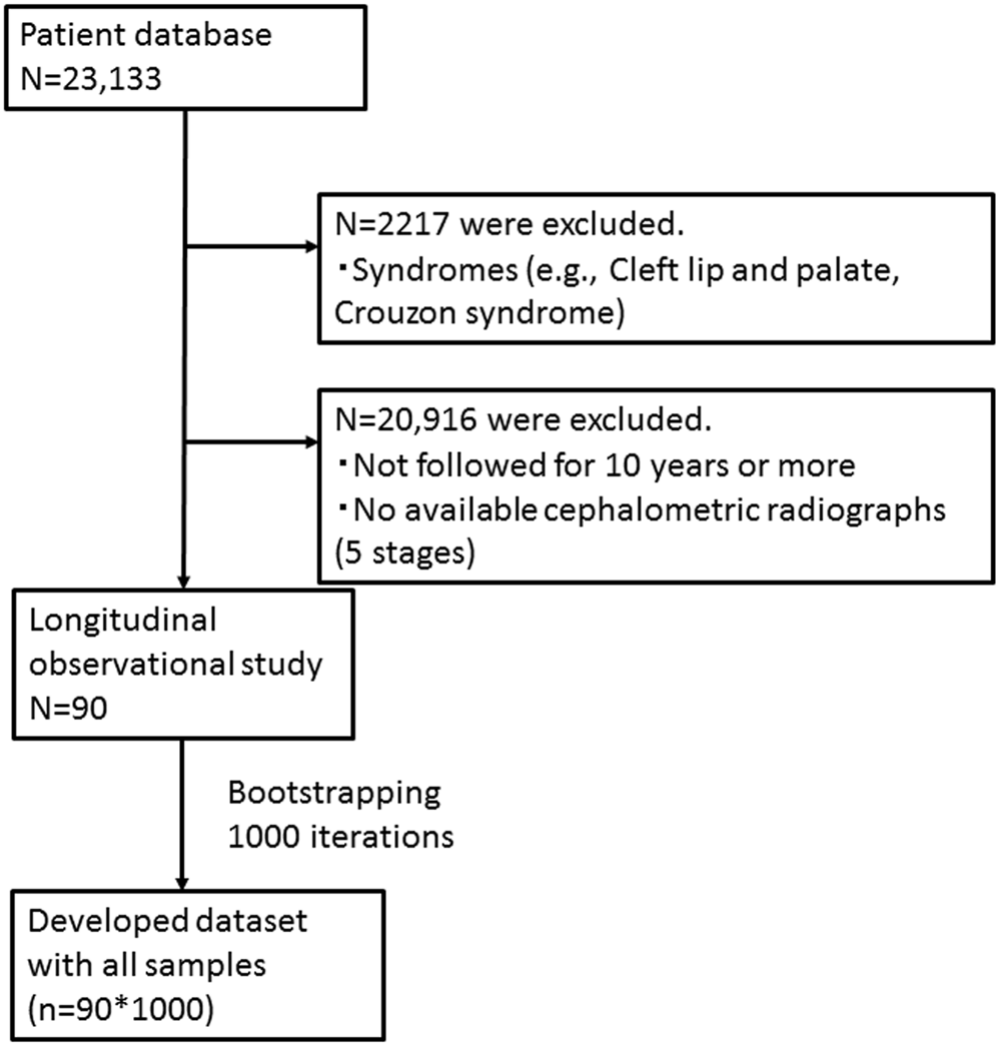
Flowchart of the longitudinal observations in subjects.

From a total of 23,133 Japanese patients who underwent lateral cephalometric radiography, 90 were retrospectively enrolled in this study (Fig. 1). The radiographs were obtained under standard conditions. Each patient’s data were evaluated at 5 stages: lower primary school (age: 8.21 ± 0.96 [mean ± standard deviation] years), upper primary school (9.91 ± 0.96 years), junior high school (12.88 ± 0.81 years), senior high school (16.01 ± 0.17 years), and young adults (18.86 ± 1.08 years).

**Figure 2:**
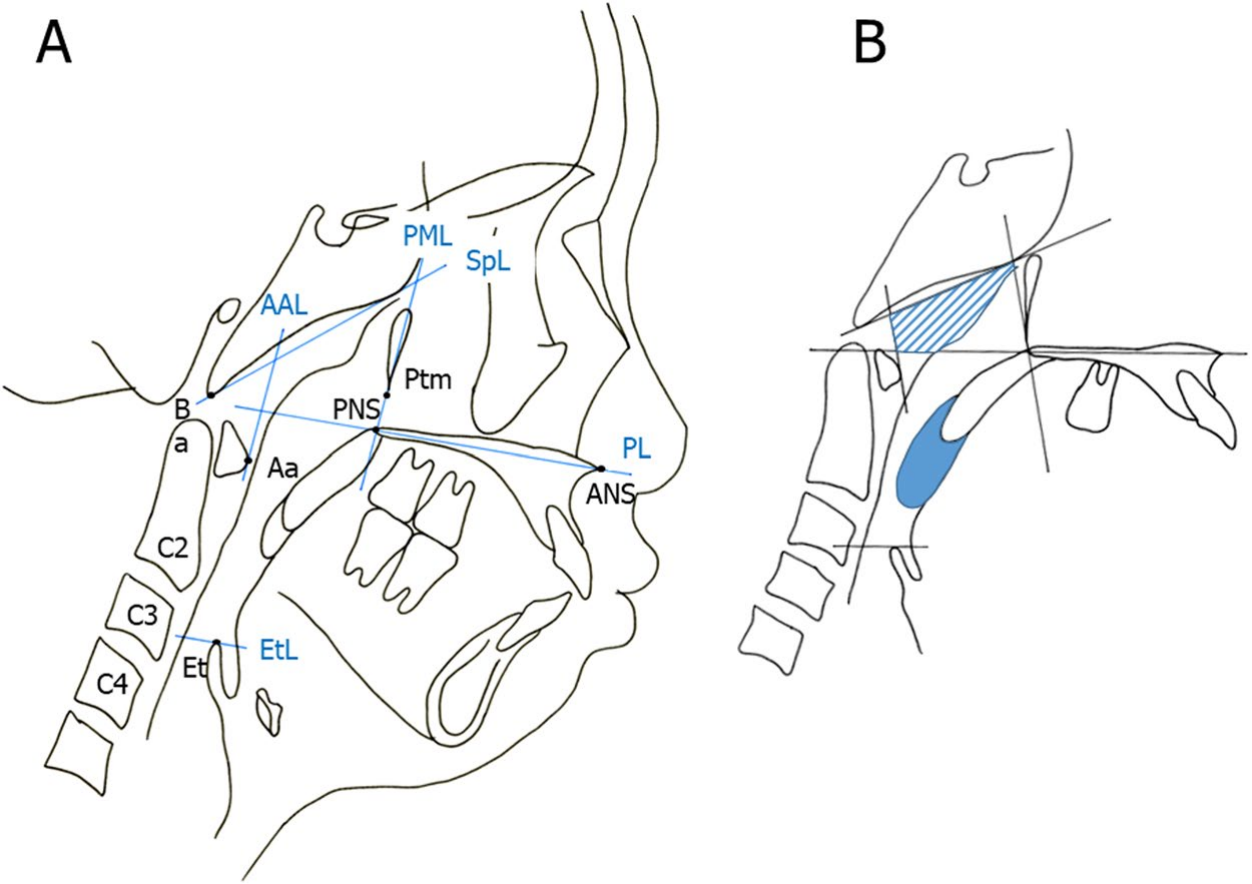
(**A**) Landmarks of the nasopharynx, oropharynx, and hypopharynx. Abbreviations: Aa, anterior medial point of the atlas; AAL, anterior atlas line (line perpendicular to the palatal line registered on the anterior medial point of the atlas); ANS, anterior nasal spine; Ba, basion; C2, second cervical vertebra; C3, third cervical vertebra; C4, fourth cervical vertebra; Et, epiglottis; EtL, epiglottis line (line parallel to the palatal line registered on the most superior point of the epiglottis); PL, palatal line (line from the anterior nasal spine to the posterior nasal spine); PML, pterygomaxillary line (line perpendicular to the palatal line registered on the pterygomaxillon); PNS, posterior nasal spine; Ptm, pterygomaxillary fissure; SpL, sphenoid line (line tangential to the lower border of the sphenoid registered on the basion). (**B**) Definitions of the adenoid (Ad) and tonsil (Tn). The partially shaded region represents the Ad area, and the part in solid colour represents the Tn area.

**Figure 3:**
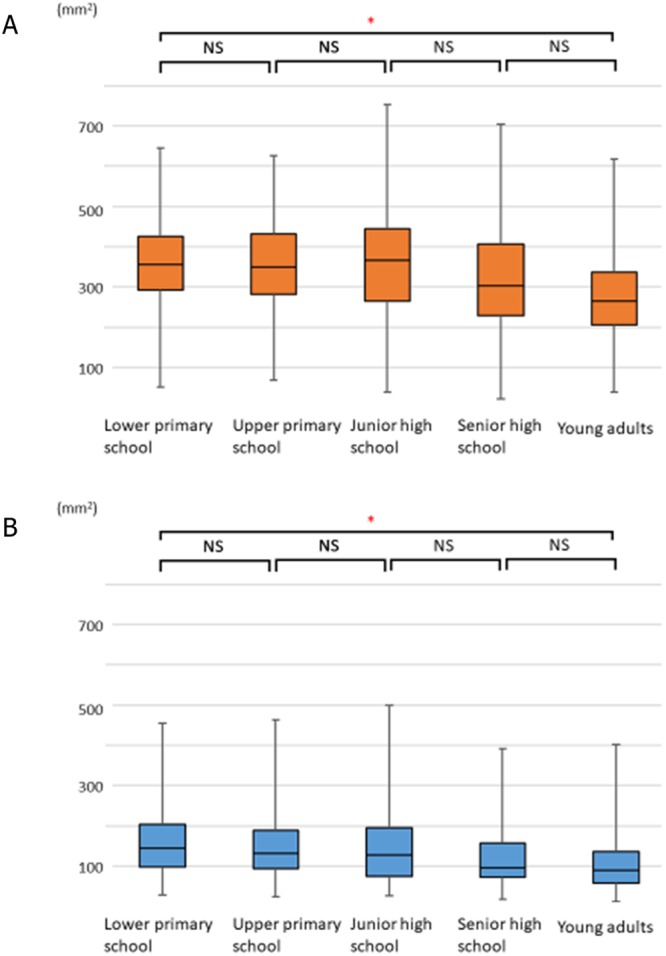
Age-dependent changes in the adenoids (Ad) and tonsils (Tn). Error bars denote 95% confidence interval determined by bootstrap analysis of 1000 iterations. (**A**) Age-dependent changes in the adenoids (Ad). A comparison of the Ad among the age groups revealed no significant changes. (347.55 ± 12.52, 346.22 ± 12.63, 355.41 ± 14.51, 316.62 ± 14.38, and 274.48 ± 13.03 mm^2^ in the lower primary school, upper primary school, junior high school, senior high school, and young adult groups, respectively). (**B**) Age-dependent changes in the tonsils (Tn). A comparison of the Tn among the age groups revealed no significant changes. (161.34 ± 9.54, 152.82 ± 9.35, 145.46 ± 9.59, 127.64 ± 8.48, 110.97 ± 8.19 mm^2^ in the lower primary school, upper primary school, junior high school, senior high school, and young adult groups, respectively).

For each group, we calculated the average cross-sectional area of the Ad and Tn using the lateral cephalometric radiographs. We divided the area into 2 parts following previously described methods^18^ (Fig. 2A,B). Briefly, the Ad area is made up of the sphenoid line (SpL) and anterior atlas line (AAL). The Tn area is outlined by the inferior border of the nasopharynx, posterior surface of the soft palate, and postero-inferior surface of the tongue^18^. The average values for the Ad were 347.55 ± 12.52 mm^2^ in the lower primary school group, 346.22 ± 12.63 mm^2^ in the upper primary school group, 355.41 ± 14.51 mm^2^ in the junior high school group, 316.62 ± 14.38 mm^2^ in the senior high school group, and 274.48 ± 13.03 mm^2^ in the young adult group. No significant decrease was observed in the Ad value across all groups. There was significant decrease between the lower primary school group and the young adult group (Fig. 3A and Table 1). Conversely, the average values for Tn were 161.34 ± 9.54 mm^2^ in the lower primary school group, 152.82 ± 9.35 mm^2^ in the upper primary school group, 145.46 ± 9.59 mm^2^ in the junior high school group, 127.64 ± 8.48 mm^2^ in the senior high school group, and 110.97 ± 8.19 mm^2^ in the young adult group. There was no significant decrease in the Tn value across all groups. There was significant decrease between the lower primary school group and the young adult group (Fig. 3B and Table 2).

**Table 1:** Mean and median size of the Ad.

Ad	Lower primary school	Upper primary school	Junior high school	Senior high school	Young adults
Mean ± s.e.m. bootstrap [mm^2^]	347.55 ± 12.52	346.22 ± 12.63	355.41 ± 14.51	316.62 ± 14.38	274.48 ± 13.03
Interquartile range [mm^2^]	98.85–178.15	110.00–184.85	122.50–241.40	144.47–220.26	95.64–209.47

**Table 2:** Mean and median size of the Tn.

Tn	Lower primary school	Upper primary school	Junior high school	Senior high school	Young adults
Mean ± s.e.m. bootstrap [mm^2^]	161.34 ± 9.54	152.82 ± 9.35	145.46 ± 9.59	127.64 ± 8.48	110.97 ± 8.19
Interquartile range [mm^2^]	143.00–180.25	66.10–127.76	96.85–146.50	73.08–121.99	59.20–99.84

The relationship between Ad and Tn among each group was analysed with simple linear regression, using Spearman’s rank correlation analysis and bootstrapping. For each age group, the correlation between the Ad and Tn sizes is shown in Tables 3 and 4.

**Table 3:** Relationship between Ad and Tn sizes using the Spearman’s rank correlation analysis.

	Correlation coefficient	*P* value
Lower primary school	0.325	0.002*
Upper primary school	0.327	0.002*
Junior high school	0.365	0.000*
Senior high school	0.302	0.004*
Young adults	0.348	0.001*

The relationship between Ad and Tn among each group was analysed using simple linear regression with Spearman’s rank correlation analysis and bootstrapping. *p < 0.05.

**Table 4:** Relationship between Ad and Tn sizes using simple linear regression analysis with bootstrapping.

	β	S.E.	*P* value	95% CI	
				Lower limit	Upper limit
Lower primary school	0.538	0.145	0.002*	0.252	0.848
Upper primary school	0.460	0.163	0.005*	0.157	0.796
Junior high school	0.566	0.171	0.002*	0.217	0.881
Senior high school	0.429	0.209	0.044*	0.076	0.913
Young adults	0.605	0.216	0.007*	0.188	1.042

The relationship between Ad and Tn among each group was analysed using simple linear regression with Spearman’s rank correlation analysis and bootstrapping. *p < 0.05.

## Discussion

In this study, we determined the average size of Ad and Tn longitudinally. Ad and Tn size did not decrease among groups (lower primary school vs upper primary school, upper primary school vs junior high school, junior high school vs senior high school, senior high school vs adult), but decreased when compared between lower primary school stage and young adult stage (a span of 15 years). Scammon plotted a ‘lymphoid-type graph’ using the weight of the thymus, which is a typical lymphoid tissue organ^1^. Our results demonstrated that the growth patterns of Ad and Tn differ from Scammon’s “lymphoid-type graph,” which described a sharp increase in size (to nearly 200%) at approximately 12 years of age and the sharp decrease by 20 years of age. Scammon’s curve of the ‘lymphoid-type graph’ has been very useful. Scammon described the developmental patterns of the thymus, pineal, pituitary, and thyroid glands and Ad as belonging to the lymphoid pattern. However, it was not clear whether the graphs for the pituitary, pineal, thyroid, and adrenal glands had specific features that differ from the typical ‘lymphoid-type graph’^1^. Hannelman *et al*. (1966) indicated that the growth and involution of the Tn and Ad, especially in the absence of recurrent bouts of tonsillitis or adenitis, generally conforms to Scammon’s curve for thymus lymph nodes and intestinal lymphoid masses^19–21^. In contrast, Pruzansky *et al*. (1975) speculated that the growth curve attributed to the Tn and Ad is not a true curve, but rather an individual response to variable stress^22^. In the present study, the sizes of the Ad and Tn were measured from the lateral cephalometric radiographs, which are standardised and highly reproducible, as demonstrated in our previous study^18^. However, it is not possible to compare our results directly with those of Scammon, who measured weight. The present findings are based on the measurement of size. Nevertheless, it is apparent that the growth patterns of the Ad and Tn differ from those of other tissues described by Scammon’s ‘lymphoid-type graph’.

According to our previous cross-sectional study, the sizes of Np and Op significantly increase with growth^18^. Although the decreases in the sizes of the Ad and Tn were small or absent, the airway occupation decreased by adulthood^18^. We believe that this is the reason why previous studies considered that the Ad and Tn completely conform to Scammon’s curve of ‘lymphoid-type graph’^20–22^.

Moreover, there was a correlation between the Ad and Tn sizes in each group. The Tn is derived from the second pharyngeal pouch, and the Ad is derived from the third pouch. The Tn has a stratified squamous epithelium, and the tonsillar crypt is deep^23–25^. In contrast, the Ad has a stratified squamocolumnar epithelium, similar to that of the nasal epithelium, which forms a well-developed barrier^23–25^. The Ad and Tn constitute Waldeyer’s ring^26^. We demonstrated that, based on direct observation of Tn in the oral cavity, the assumed size of Ad could be scientifically proven with significance.

Tonsillectomy was first described 3000 years ago^27,28^. Adenotonsillectomy is one of the most common surgical procedures. Ogra *et al*. (1971) reported that tonsillectomy and adenoidectomy reduce polio virus antibody levels and antibody responses^29^. It has also been reported that there are transient changes in cell-mediated and humoral immunity, within the normal range, after tonsillectomy, but no clinically significant differences were observed in terms of an increased risk of infection in long-term observations^30^. The indications for adenotonsillectomy are infection (e.g., recurrent tonsillitis, chronic tonsillitis, and peritonsillar abscess) and upper airway obstruction caused by hypertrophy of the lymphatic tissue in Waldeyer’s ring. However, various criteria are used in different countries^13,14,31^. The usefulness of adenotonsillectomy was described in the 2002 clinical guidelines for diagnosis and treatment of paediatric OSA syndrome by the American Academy of Pediatrics^11^. In 2011, the American Academy of Otolaryngology—Head and Neck Surgery presented guidelines for adenotonsillectomy in children^32^. The results of the present study can be used as a basis for size evaluation, which is a criterion for choosing adenotonsillectomy. It can facilitate determination of the optimum time for adenotonsillectomy and serve as a criterion for deciding upon the removal of both Ad and Tn, or only one of these.

In addition, our previous study suggested that the growth of the airway continues until about 12 years of age^18^. Orthodontists often use functional appliances, such as the twin block (TB) or maxillary protracting appliance (MPA), for patients with skeletal retrognathia or maxillary hypoplasia who are in their peak growth periods. Such functional appliances are effective and are widely used for promoting skeletal growth in orthodontic treatment during the growth period^33^. A previous study reported that after the use of TB in growing children, the upper airway showed a significant enlargement of the Np, Op, and hypopharynx^34^. Another study reported that use of an MPA increased the upper airway dimensions^35^. Therefore, orthodontic treatment is likely to contribute not only to improving the facial profile, but also to improving the fundamental and essential functions of the respiratory system. It is, thus, important in children.

Similar to our previous study, in this study, we examined 90 cases from 23,133 Japanese patients who underwent lateral cephalometric radiography between 1987 and 2018. Although more intensive than cross-sectional studies, longitudinal observational studies are more suitable for assessing complex growths in individuals. Cephalometric radiography is a routine examination in orthodontic diagnosis and treatment; hence, the patients in this study did not require additional radiological exposure. Standardised lateral cephalometric radiographs are highly reproducible because the source-to-subject-to-film distances remain fixed, and it is possible to calculate the subject size from the magnification of the film^36–38^. A previous study reported that evaluation of the 2-dimensional upper airway area by lateral cephalometric analysis correlates well with the 3-dimensional upper airway assessment, and it can be used as a screening test to predict the airway volume by computed tomography^39^.

The growth patterns of the Ad and Tn differ from Scammon’s ‘lymphoid-type graph’. The present findings may be helpful for predicting the growth and development of the Ad and Tn.

## Methods

All experiments were performed in accordance with the relevant guidelines and regulations. Ninety Japanese children (31 boys and 59 girls; age, 6–20 years) were selected from 23,133 Japanese patients who underwent lateral cephalometric radiography. Exclusion criteria were the presence of syndromes and previous adenoidal and tonsillar surgeries (Fig. 1).

For the measurement of Ad and Tn, all cephalometric radiographs were obtained using the commonly used international standard settings^36–38,40^. All cephalometric radiographs were obtained with the head positioned in a cephalostat, with the head fixed with the ear rods, and patient’s Frankfurt plane set to be parallel to the floor. The source-to-subject and subject-to-film distances were always fixed. Therefore, it was possible to evaluate the developmental change over time during the growth period^18^. (In Japan, when obtaining a cephalometric radiograph, the distance from the film to the patient’s midsagittal plane is set to 15 cm, and the distance from X-ray source to the film is 165 cm). Cephalometric landmarks used in this study are shown in Fig. 2A.

Using WinCeph Version 9.0 (Rise Corp., Tokyo, Japan), the areas were measured 3 times on 3 days, and the average value of each area was calculated. Lateral cephalometric tracing and analysis were performed by the same investigator. On average, the random method errors for the size of Ad and Tn were 1.43 mm^2^ and 1.15 mm^2^, respectively^41^.

### Study Approval

This longitudinal observational study was approved by the Research Ethics Committee (Permission number: D2015-626) of Tokyo Medical and Dental University Dental Hospital (Tokyo, Japan). Informed consent was obtained from either the study patients or their parents.

### Statistical Analysis

All statistical analyses were performed using SPSS statistics (Version 25.0, IBM, NY, USA). The average, standard errors, and 95% confidential interval (CI) of each group were estimated using bootstrapping with 1000 iterations. The Kruskal–Wallis test was used (*P* < 0.01) to evaluate size differences of Tn and Ad among the age groups. When a significant difference was observed, the significance of the difference was assessed using the Steel–Dwass test.

The relationship between Ad and Tn among each group was then analysed using simple linear regression with Spearman’s rank correlation analysis and bootstrapping. Since the sample size of the present study was small, the 95% CI was estimated using bootstrapping.

## Electronic supplementary material

Supplementary information
