# Supplementary material for: Patterns of adenoid and tonsil growth in Japanese children and adolescents: A longitudinal study
Source: Sci Rep. 2018 Nov 20;8:17088. doi: 10.1038/s41598-018-35272-z (PMC6244207; doi:10.1038/s41598-018-35272-z)

**Appendix**

**Patterns of adenoid and tonsil growth in Japanese children and adolescents: A longitudinal study**

Takayoshi Ishida*^1^, Asuka Manabe^1^, Shin-Sheng Yang^1^, Hyung Sik Yoon^2^, Eiichiro Kanda^3^, and Takashi Ono^1^

^1^ Department of Orthodontic Sciences, Tokyo Medical Dental University Graduate School, Tokyo, Japan

^2^ All Barun Dental Clinic, Suwon, Korea

^3^ Department of Nephrology and Hypertension, Tokyo Kyosai Hospital, Tokyo, Japan

***Corresponding author:** Takayoshi Ishida

Department of Orthodontic Sciences, Tokyo Medical Dental University Graduate School, Tokyo 113-8549, Japan

Fax & Tel: +81-3-5803-4146

E-mail: [takayoshiishida@gmail.com](mailto:takayoshiishida@gmail.com)

*Relationships between adenotonsillar size and orthodontic abnormalities*

The subjects consisted of 90 lower primary school Japanese.

It was found that there was a significant positive correlation between the size of the Ad/Tn and facial height (i.e., the distance between Nasion and Menton).

The relationships were analyzed with the simple linear regression, using Spearman’s risk correlation and bootstrapping.


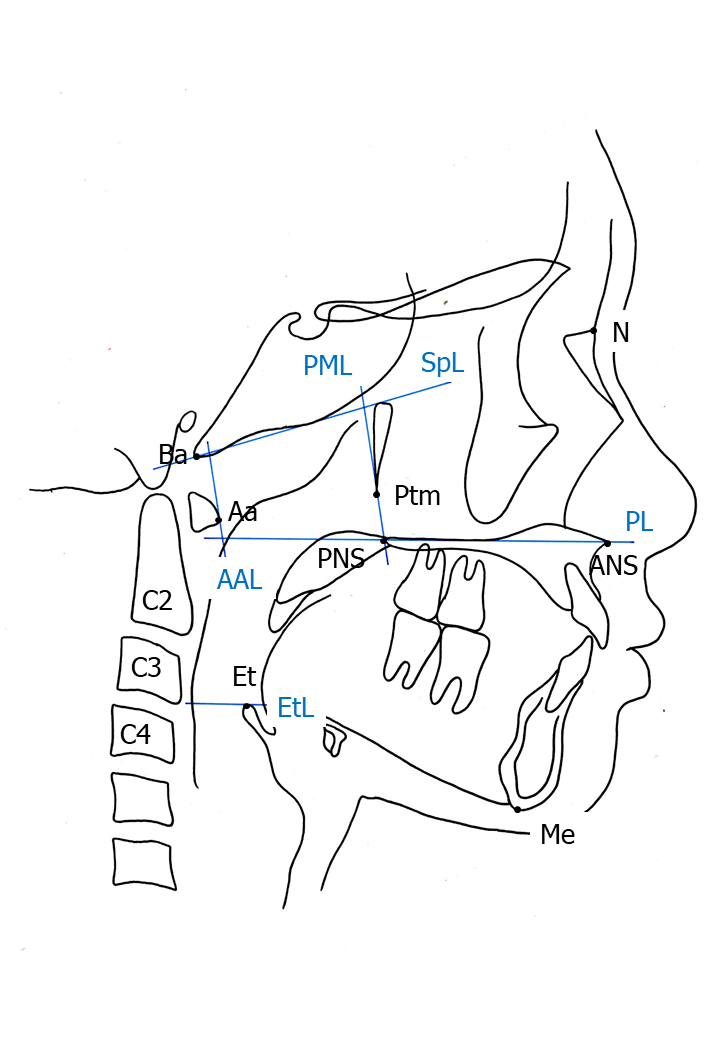


FH: The distance between N and Me


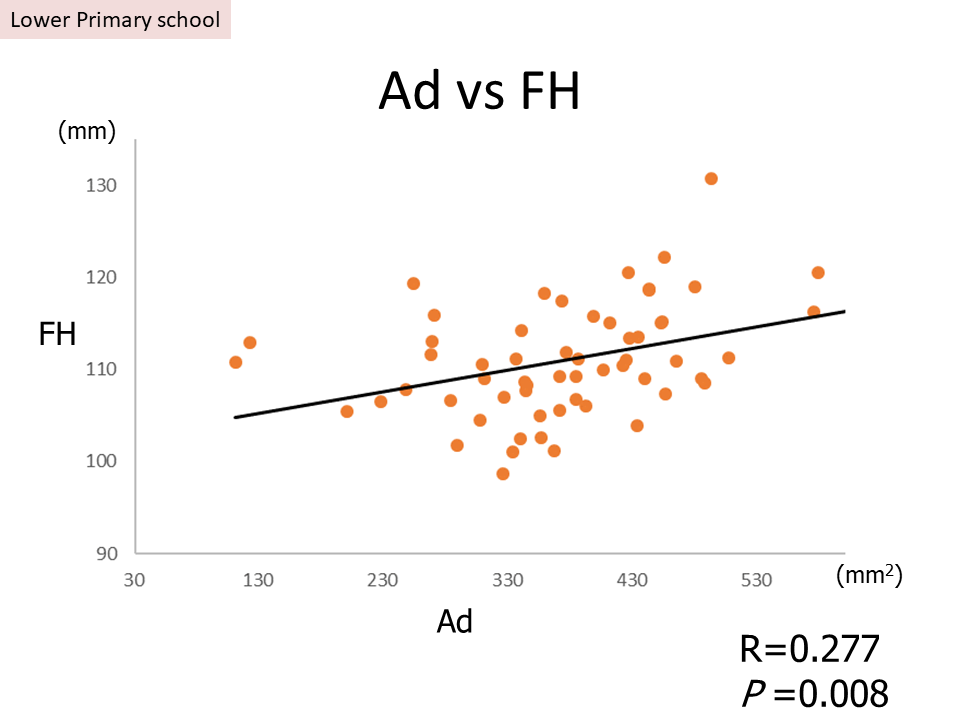


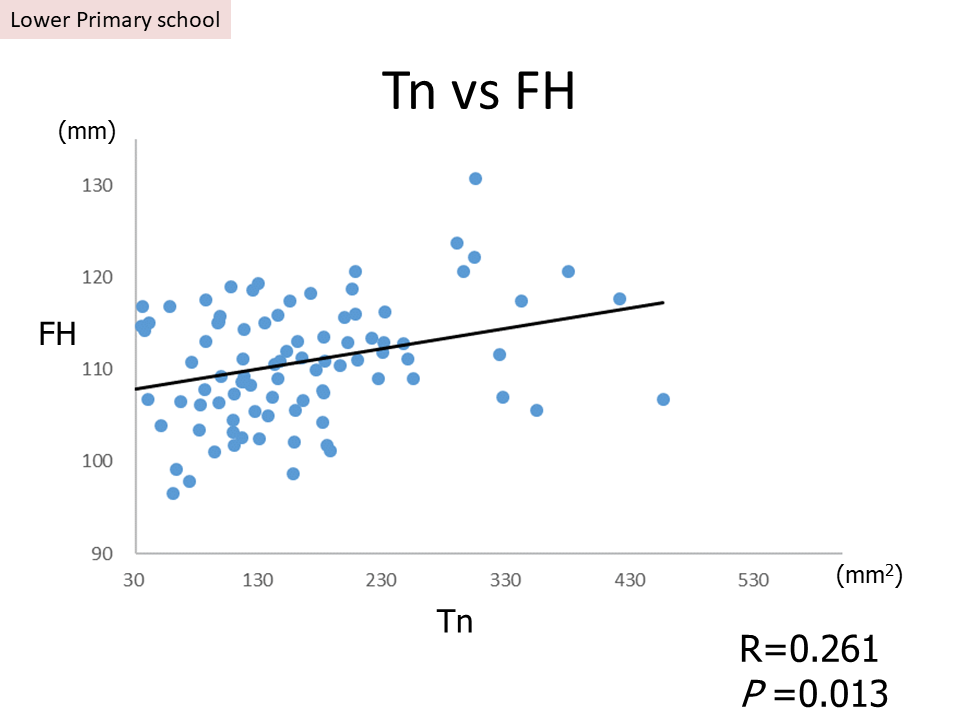

Supplement: Supplementary file 1 — Supplementary information [file 41598_2018_35272_MOESM1_ESM.docx]
